# Preclinical efficacy of oral and nasal rivastigmine-loaded chitosan nano-particles on AlCl_3_-induced Alzheimer’s-like disease in rats

**DOI:** 10.1007/s10787-024-01541-9

**Published:** 2024-08-11

**Authors:** Dina E. ElMosbah, Marwa S. Khattab, Marwa A. Ibrahim, Mona I. El-Asssal, Hala M. F. El Miniawy

**Affiliations:** 1 Department of Pathology, Faculty of Veterinary Medicine, Cairo University, Giza, 12211 Egypt; 2 Department of Biochemistry and Chemistry of Nutrition, Faculty of Veterinary Medicine, Cairo University, Giza, 12211 Egypt; 3 Department of Pharmaceutics and Pharmaceutical Technology, Faculty of Pharmacy, Future University in Egypt, Cairo, 11835 Egypt

**Keywords:** Alzheimer's disease, Rivastigmine nanoparticles, Histopathology, Tau, Caspase-3, Nrf-2

## Abstract

The successful treatment of Alzheimer’s disease (AD) is still a big challenge. Rivastigmine is one of the most used drugs for the treatment of AD. The short half-life, lower bioavailability, and less concentration of the drug in the brain after oral delivery are considered the main drawbacks of rivastigmine. To improve these drawbacks, nanostructure-mediated drug delivery has gained more attention. This study investigates the effect of rivastigmine-loaded in optimized chitosan nano-particles (RS-CSNPs) as polymeric nano-carriers by different administration routes (oral and intranasal) on aluminum chloride (AlCl_3_)-induced Alzheimer-like disease in rat. The model was established by giving rats 100 mg/kg/b.wt of AlCl_3_ orally for 3 months. Then the experimental rats were treated with RS-CSNPs either orally or intranasally for 75 days. Histopathology, immunohistochemistry of Tau expression in brain tissue, and gene expression of Caspase-3, NF-κB, and Nrf-2 were carried out. The therapeutic agents used decreased the alterations observed in AlCl_3_ group with improvement in the neuronal viability. In addition to low expression of tau protein, down-regulation of caspase-3 and NF-κB genes and up-regulation of Nrf-2. RS-CSNPs alleviated the progression of AD presumably via blocking the inflammatory cascade and decreasing the oxidative stress process. The intranasal route is superior to the oral one and promising in AD management.

## Introduction

Up till now, there is no effective therapy for AD. Current treatments available include Food and Drug Administration (FDA)-approved drugs, such as rivastigmine, galantamine, memantine, and donepezil. However, they only improve quality of life when prescribed at the appropriate time (Botchway et al. 2018; Sabandal et al. 2022).

Rivastigmine is the most widely used as it inhibits both acetylcholinesterase (AChE) and butyrylcholinesterase. It has selectivity for the hippocampus and cortex rather than the striatum and pons/medulla in the brain (Farlow. 2003). It is found in both oral formulations and transdermal patches. The traditional form of rivastigmine has numerous drawbacks like high first pass metabolism, short half-life, and lower bioavailability. Lesser concentration of the drug in the brain after oral delivery necessitates frequent dosing which results in severe cholinergic side effects, such as bradycardia, nausea, vomiting, anorexia, and fatigue (Briggs et al. 2016; Mohammad et al. 2017).

Delivery of drugs to the brain is the most challenging part of treating neurological disorders due to the interference of blood–brain barrier (BBB) for the movement of some molecules in the brain parenchyma (Mikitsh and Chacko. 2014). The use of nanotechnology can improve the bioavailability and specificity of the drug targeting the brain (Tosi et al. 2013; Harilal et al. 2019).

Unlike oral route, the intranasal (I/N) route provides a convenient non-invasive and rapid route for the direct delivery of drugs from the olfactory region of the nasal passages into brain by passing the BBB (Illum. 2000). This route leads to low systemic side effects, enhanced bioavailability, and higher therapeutic efficacy. However, few setbacks, such as mucociliary clearance and poor drug mucosal permeation, reduce their absorption rate. For this reason, its translation from the laboratory to the clinic is limited. To overcome this obstacles, suitable nano-carriers should be loaded with drugs which may lead to prolonged delivery of drugs with better permeability and high efficacy like chitosan (Rassu et al. 2016; Abdou et al. 2017). Researchers have formulated rivastigmine-loaded nanoemulsion which revealed significantly higher drug concentration in brain than the solution (Haider et al. 2018). Previous pharmacokinetic study indicated that rivastigmine-loaded chitosan nano-particles administered intranasally have higher concentration in brain with better brain targeting (Fazil et al. 2012).

Although extensive research has been conducted to advance AD therapy from the laboratory to the clinic, no nano-carriers have hit the market yet. Since nanotechnology has only been studied in preclinical trials, further work is still required to pass to clinical trials. In this study, we investigate the therapeutic effect of oral and I/N rivastigmine-loaded optimized chitosan nano-particles on Alzheimer's-like disease induced by AlCl_3_ in rats.

## Materials and methods

### Animals

Thirty adult male albino rats weighing 200–250 g (VACSERA Laboratory Animal House, Giza, Egypt) were kept for 7 days to acclimatize. Plastic cages and sawdust bedding were used for housing at 25 ± 2 °C in a 12/12 h light–dark cycle with food ad libitum and free access to water. Any distress and behavioral changes were recorded daily. Isoflurane was used to tranquilize animals during blood sampling. The experiment was conducted in the Pathology department, Faculty of Veterinary Medicine, Cairo University.

## Preparation of rivastigmine-loaded nano-particles

Desperation of rivastigmine (RS) loaded in optimized chitosan nano-particles (CSNPs) as polymeric Nano-carriers was prepared. Chitosan nano-particles were prepared using ionotropic gelation of chitosan with sodium tripolyphosphate STPP anions (Zynopsicha et al. 2019) with some modification. In brief, chitosan was dissolved in purified water at various ratios 0.5% w/v, 0.7% w/v, 1% w/v, 1.2% w/v, and 1.4% w/v. STPP was also dissolved in purified water at various concentrations of 0.6% w/v, 0.8% w/v, 1% w/v, 1.2% w/v, and 1.4% w/v. Each ratio of chitosan was dissolved in aqueous solution containing 1% (v/v) glacial acetic acid and leaving it under high stirring (1400 r/min) for 2 h. The pH was adjusted to pH 5 with 0.1N NaOH. Tween 80 was added as modified ingredient with constant ratio 0.2 ml. STPP as a physical cross-linker was dissolved separately in deionized water and added to the chitosan solution as drops at high rate under vigorous magnetic stirring at room temperature (25 °C) (Liu et al. 2007). Nanoparticles formed spontaneously under mechanical stirring, the resulting aqueous dispersion was then homogenized (Heidolph, PH-E3.7-HGZI, Germany) at 22,000 r/min for 2 min and left under ultra-sonication for 5 min. Different formulae were prepared with different STPP: chitosan ratios. Desirability factor excel sheet was used, plain nano-particles formulae data were applied to choose the optimal formula depending on the smallest particle size, the lowest PDI and the highest zeta potential. The choice of the optimal formula and the in vitro evaluation parameters of optimized loaded RS-CSNPs desperation have been explained in previous published research (El-Assal and Samuel. 2022).

## Experimental design

Thirty adult male albino rats were assigned into 4 groups as follows: GI rats were orally administered distilled water daily and served as control negative group. GII (oral RS-CSNPs group), rats were orally administrated with desperation of RS-CSNPs at a dose of 1mL (containing 0.13 mg Rivastigmine). GIII (nasal RS-CSNPs group), rats were intranasally administrated with desperation of RS-CSNPs. GIV rats were orally administrated AlCl_3_ (100 mg/kg/b.wt daily) for 90 days to induce AD (Singh et al. 2018; ElMosbah et al. 2024). After that, GIV were subdivided into 3 subgroups of 5 rats each as follows: GIVa (AlCl_3_ group), kept as positive control group, GIVb (AlCl_3_ + oral RS-CSNPs group), rats were treated orally with 1mL RS-CSNPs (containing 0.13 mg Rivastigmine) daily for 75 days (Kumar and Kumar. 2009), GIVc (AlCl_3_ + nasal RS-CSNPs group), rats were treated with RS-CSNPs by intranasal route daily for 75 days.

## Histopathological examination:

Specimens from brain, nasal mucosa, stomach, intestine, and liver were collected and preserved in 10% neutral buffered formalin for fixation, processed by ascending concentration of ethanol and xylene, and embedded in paraffin. A rotary microtome (RM2135; Leica, Wetzlar, Germany) was used for tissue sectioning (3–4 µm thick). Brain sections were stained with hematoxylin and eosin (H&E) (Bancroft and Gamble. 2013). Special stain as Cresyl violet (Nissl) stain was used for observation and quantification of neurons in the cerebral cortical regions. For cells quantification, high-quality images were captured at 400 × magnification of light microscopy (BX50F4; Olympus, Tokyo, Japan). Neurons with visible nuclei and well-defined edges which were considered viable were counted. The viability cell percent was calculated from the following equation

$$\text{The viability cell percent }(\text{\%})=\frac{\text{viable neuron count}}{\text{Total neuron count}}\times 100$$

## Immunohistochemistry (IHC)

The expression of phosphorylated tau as a marker of AD severity was evaluated in paraffin tissue sections of all groups using the immunoperoxidase technique. Briefly, tissue sections were deparaffinized, rehydrated, and underwent antigen retrieval using sodium citrate buffer. They were then incubated with 1:100 phosphorylated tau (abx328322, Abexxa, UK) followed by the 2ry biotinylated antibody and later by the avidin peroxidase complex (Vactastain ABC peroxidase kit, Vector Laboratories, Burlingame, CA, USA). Color development was performed by chromogen 3, 3-diaminobenzidine tetrahydrochloride (DAB, Sigma Chemicals, Perth, Australia). Slides were counterstained by hematoxylin and then examined under a light microscope (Magaki et al. 2019).

## Gene expression analysis

Total RNA was extracted using the Qiagen mini-RNeasy extraction kit, following the manufacturer's instructions. The concentration and the purity of the extracted RNA were determined using spectrophotometry at wavelengths 260 and 280 nm (Farid et al. 2023). Subsequently, complementary DNA (cDNA) was synthesized using the RevertAid First Strand cDNA Synthesis Kit (Thermo scientific) according to the manufacturer's guidelines, after treating the samples with DNase I to remove DNA contamination (Fermentas, Lithuania).

To detect the mRNA levels of specific genes, primer sets were designed using the Rattus Norvegicus sequences available in GenBank (Table 1). The relative expression of the target genes was evaluated using real-time PCR analysis and SYBR Green PCR Master Mix. The real-time PCR was performed on the ABI Prism Step One Plus Real-Time PCR System from Applied Biosystems, following the manufacturer's instructions. Each sample was subjected to two PCR runs. The expression levels of the studied genes were normalized to the housekeeping gene beta-actin (*β-Act*) (Ahmed et al. 2022). The gene expression data were calculated using the DDCt technique.

**Table 1:** The sets of primers used in the study

Gene	Forward	Reverse	Product size	Accession number
NF-κB	ACCTGGAGCAAGCCATTAGC	AGTTCCGGTTTACTCGGCAG	234	NM_199267.2
Nrf-2	TGTAGATGACCATGAGTCGC	TCCTGCCAAACTTGCTCCAT	159	NM_031789.2
Caspase-3	CATGCACATCCTCACTCGTG	CCCACTCCCAGTCATTCCTT	158	NM_012922.2
β-Act	CCGCGAGTACAACCTTCTTG	CAGTTGGTGACAATGCCGTG	297	NM_031144.3

## Results

### Histopathology

#### Brain

**Fig. 1:**
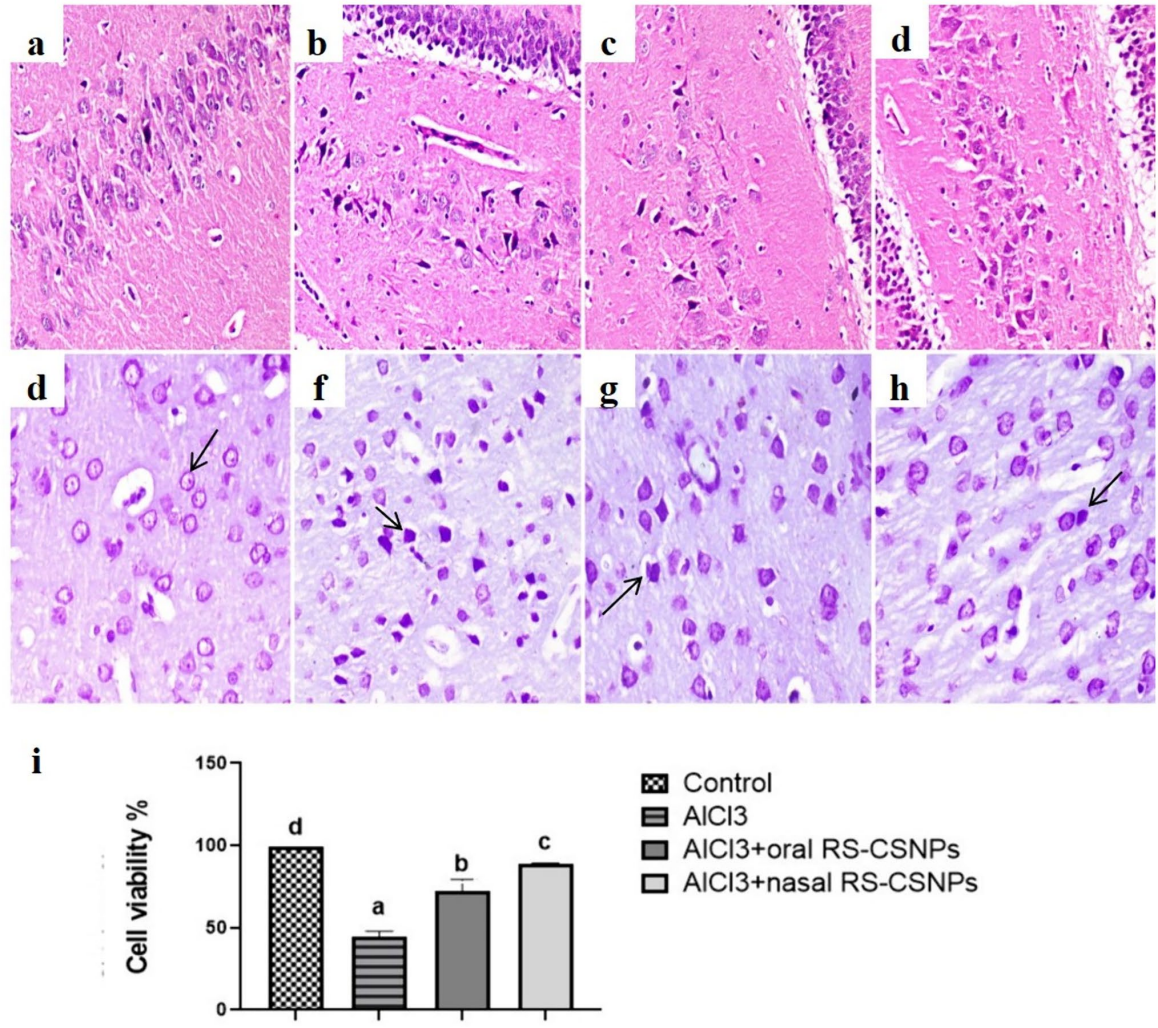
Photomicrographs of rat brain, hippocampus region (H&E) stained; **a** Normal structure of CA3 region in Control group, **b** Numerous degenerating neurons within the CA4 region with angiopathy in AlCl3 group. Decrease of cell density noticed, **c** Moderate neuronal degeneration in CA4 region in AlCl3 + oral RS-CSNPs group, (d) Mild neuronal degeneration in CA4 region in AlCl3 + nasal RS-CSNPs group, 200x. Photomicrographs of the cerebral cortex in different groups of rats stained with Cresyl violet (Nissl) stain. **e** Neurons have visible spherical nucleus and well-defined border in control group (arrow), **f** Many shrunken, deformed, and hyperchromatic dead neurons with indistinct borders (arrow) in AlCl3 group, **g** Reduced neuronal degeneration in AlCl3 + oral RS-CSNPs group, and **h** in AlCl3 + nasal RS-CSNPs group, 400x. **i** The cell counts and percent of viable neurons after cresyl violet staining were significantly decreased in AlCl3 group indicating a higher neuronal loss compared to other groups. All data presented as mean ± SE. Values bearing different lowercase letters (**a**,**b**,**c**,**d**) are significant at *P* values less than 0.05

The control group brain was characterized by normal histological structure of different regions (Fig. 1a). No histological alteration was observed in oral RS-CSNPs and nasal RS-CSNPs group. After stopping the administration of AlCl_3_ during treatment period, the brain lesion continued and progressed. The most pronounced lesion observed in the cerebral blood vessels was severely thickened walls (cerebral angiopathy) with endothelial capillary proliferation in some of them in addition to presence of hemorrhages which were mainly observed in cerebral cortex, striatum, and mid-brain region. Pink material was deposited with astrocytes infiltrations around some blood vessels. Neuronal degeneration and neuronophagia accompanied with marked astroglial and microglial reactions (rod cells) were observed. Abnormally appearing cells with flame shaped processes were observed especially in CA3 and CA4 hippocampal regions with decreased cellular density (Fig. 1b). Vacuolization of neuropil was more intense in striatum region. On the other hand, rats treated with oral and nasal RS-CSNPs exhibited moderate alteration with no vascular injury or hemorrhage (Fig. 1c, d). However, congestion of cerebral cortex was a common finding.

The viability of neurons in cerebral cortex regions was evaluated using Cresyl violet (Nissl) staining. The viable normal neurons appeared with visible nuclei and well-defined edges while the dead (degenerated) neurons were dark bluish-stained, shrunken, deformed, having indistinct borders between the nucleus and cytoplasm (Fig. 1e-h). Viable neurons % were significantly reduced indicating higher neuronal loss and degeneration in the brain of rats receiving AlCl_3_ in comparison to other groups. There was a significant increase in cell viability % and less neuronal degeneration in AlCl_3_ + nasal RS-CSNPs group compared to AlCl_3_ + oral RS-CSNPs (Fig. 1i).

#### Nasal cavity

**Fig. 2:**
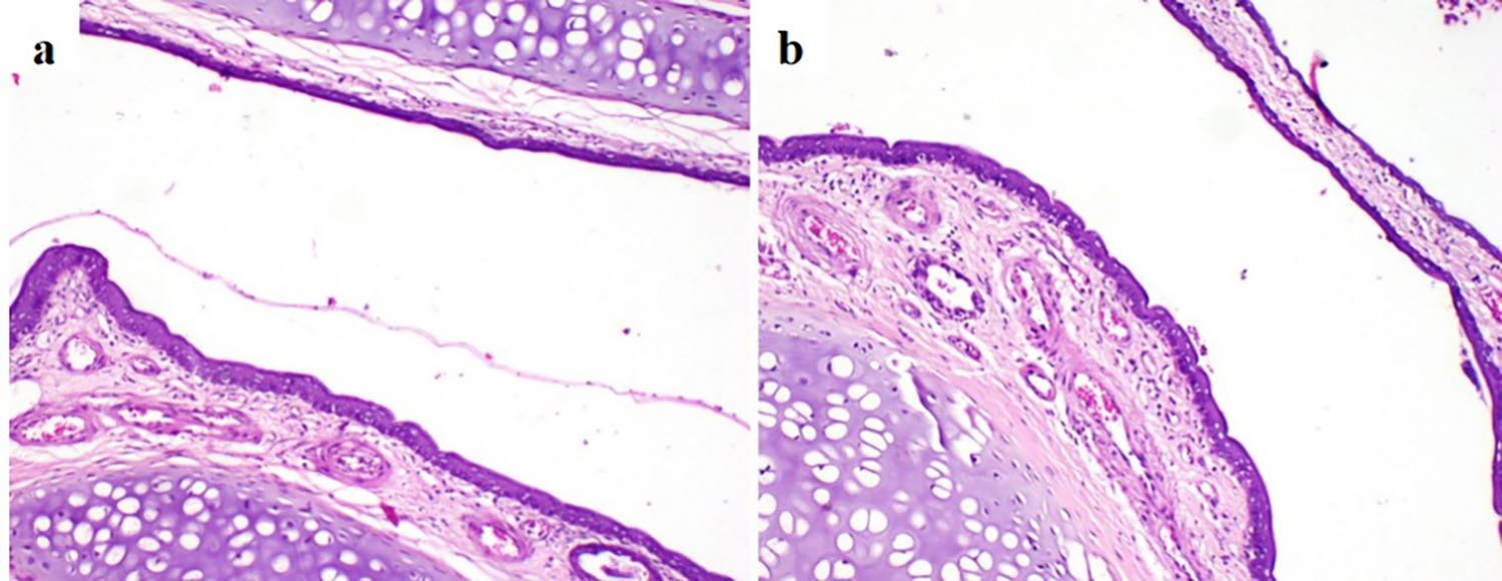
Photomicrographs of rat nasal cavity (H&E) stained; **a** Normal structure of nasal mucosa in Control group, **b** Mild inflammatory cell infiltration in lamina propria with blood vessels congestion in nasal RS-CSNPs group, 100x

Control group and oral RS-CSNPs group, nasal mucosa revealed normal structure with intact basement membrane and no mucociliary damage (Fig. 2a). Focal ulcerated area noted in some parts of the nasal mucosa with inflammatory cell infiltration in lamina propria in AlCl_3_ + nasal RS-CSNPs group, while rats receiving RS-CSNPs intranasally showed mild histological alteration in nasal mucosa which was characterized by thickening of lamina propria by mild inflammatory cell infiltration and congestion of blood vessels (Fig. 2b).

#### Stomach, intestine, and liver

Microscopic examination of the tissue sections of stomach, intestine, and liver of rats in control group and AlCl_3_ + nasal RS-CSNPs group showed normal histological structures. In oral RS-CSNPs group, there was hyperplasia of gastric gland lining epithelium with focal mild necrosis of mucosal surface and mononuclear inflammatory cells infiltration in the gastric submucosa. The intestine microscopy revealed mild necrosis of enterocytes and goblet cell hyperplasia with excessive mucous accumulation in the lumen and between villi in addition to mononuclear inflammatory cells infiltration in lamina propria and submucosa. In AlCl_3_ group, there were mucous exudation, mild sloughing of mucosal surface and mononuclear cells infiltration in the gastric submucosa in addition to desquamation of the enterocytes, necrosis of intestinal glands and heavy infiltration of mononuclear inflammatory cells in lamina propria and submucosa. In AlCl_3_ + oral RS-CSNPs, the stomach and intestinal lesions were similar to those observed in AlCl_3_ group and oral RS-CSNPs group but more severe.

**Fig. 3:**
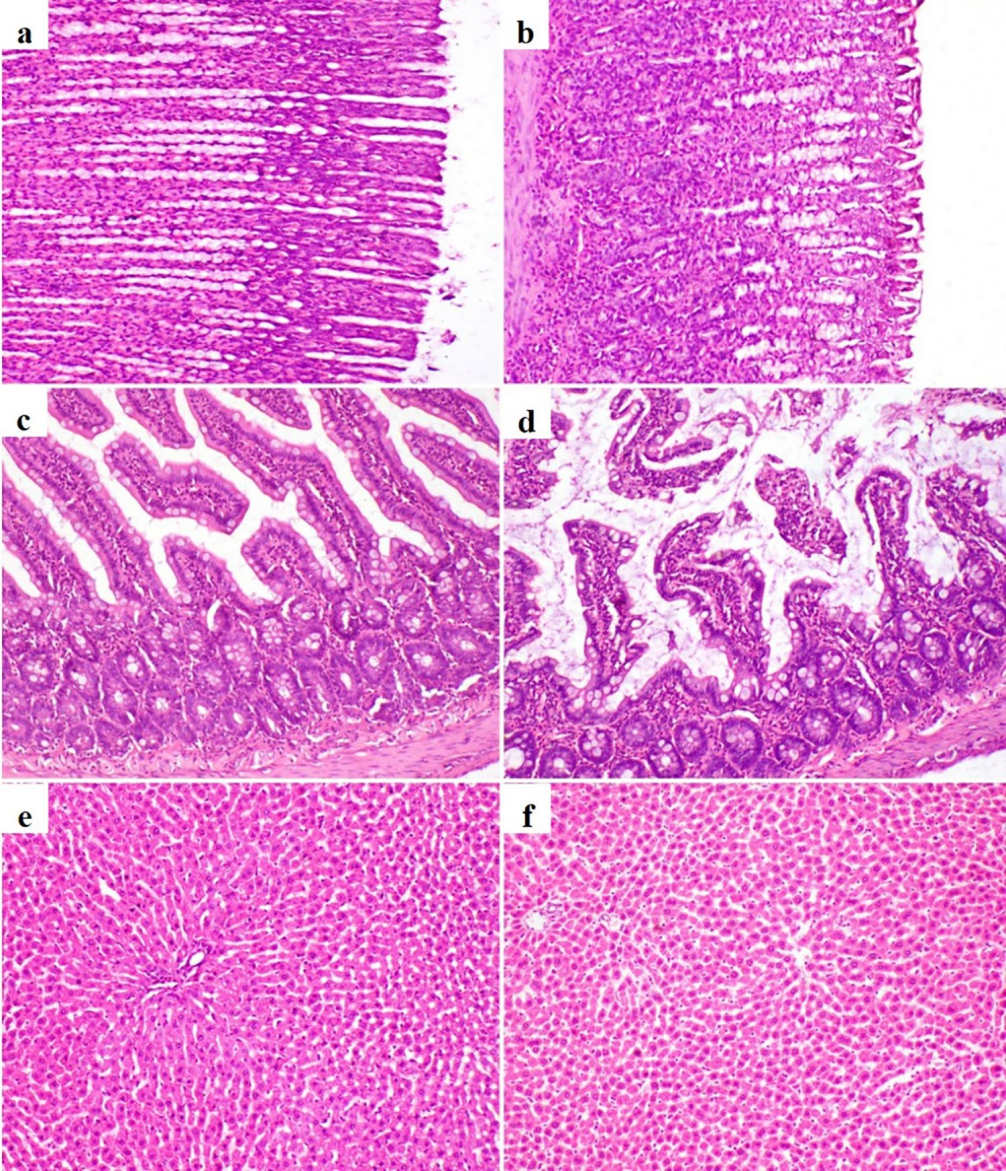
Photomicrographs of rat stomach, intestine and liver (H&E) stained; **a** Normal structure of gastric mucosa in control group **b** Mild mononuclear cells infiltration in the gastric submucosa in oral RS-CSNPs group, **c** Normal structure of intestinal mucosa in control group, **d** Mild mucous exudation with goblet cell hyperplasia of intestinal mucosa in oral RS-CSNPs, **e** Normal hepatic structure in control group, **f** Mild hepatic dissociation with hyperplasia and hypertrophy of Kupffer cells in oral RS-CSNPs group, 100x

In oral RS-CSNPs, the liver microscopy showed mild hepatic dissociation with hyperplasia and hypertrophy of Kupffer cells in (Fig. 3). In AlCl_3_ group and AlCl_3_ + oral RS-CSNPs group, hepatocellular injuries with multifocal to diffuse vacuolar degeneration, leukocytosis, and periportal inflammation were seen.

#### Immunohistochemistry investigation

**Fig. 4:**
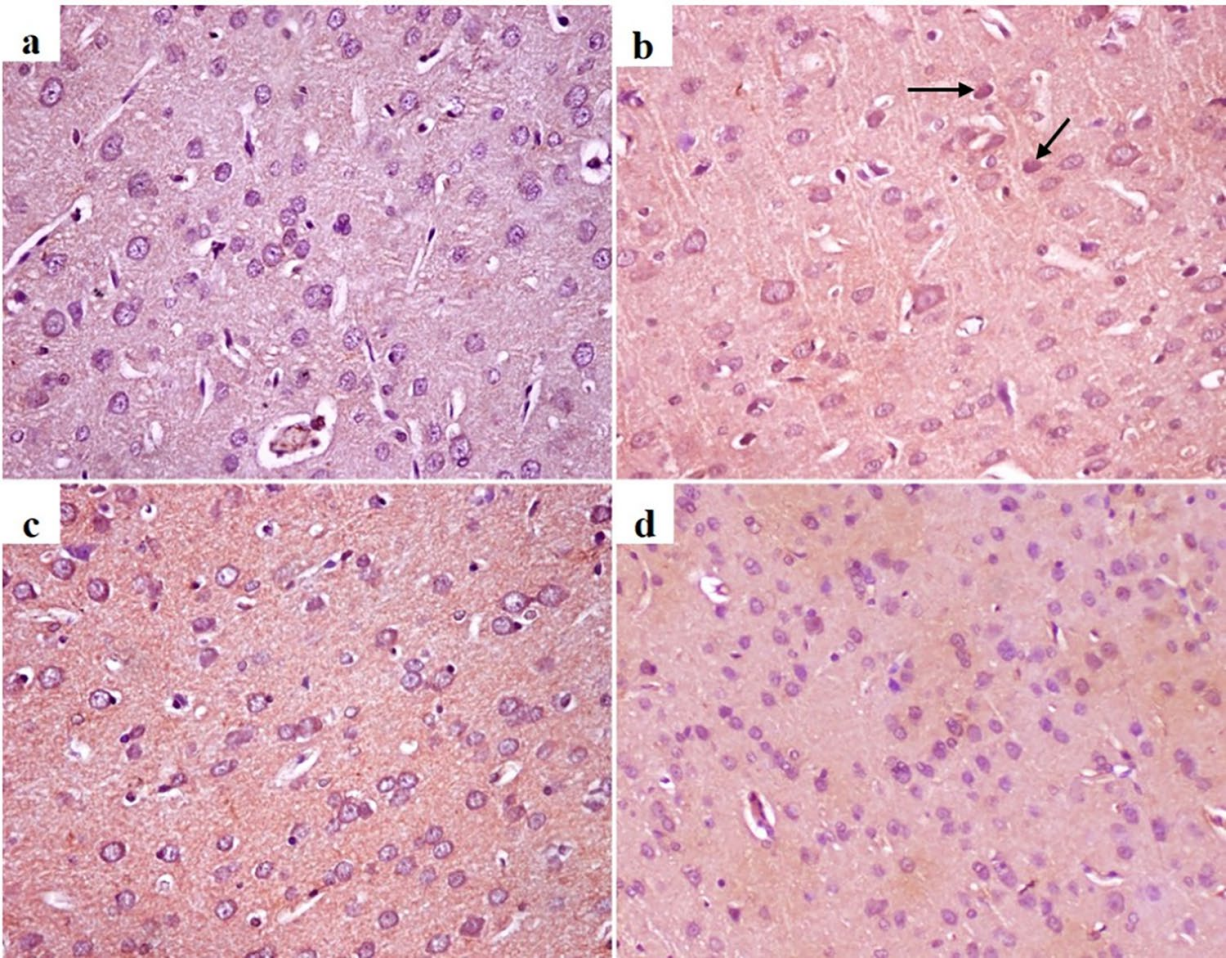
Immunohistochemistry of Tau expression in brain tissue in different groups. (**a**) Absence of Tau expression in control group, (**b**) Severe expression of Tau in neurons of cerebral cortex in AlCl3 group (arrow), (**c**) and (**d**) Mild expression of Tau in AlCl3 + oral RS-CSNPs group and AlCl3 + nasal RS-CSNPs group respectively, 200x

Tau expression was widely expressed in neurons in different brain regions. There is no expression in control group. The severe expression was observed in AlCl_3_ group. On the other hand, it was mild in AD induced rats treated with RS-CSNPs (Fig. 4).

#### Genetic analysis

**Fig. 5:**
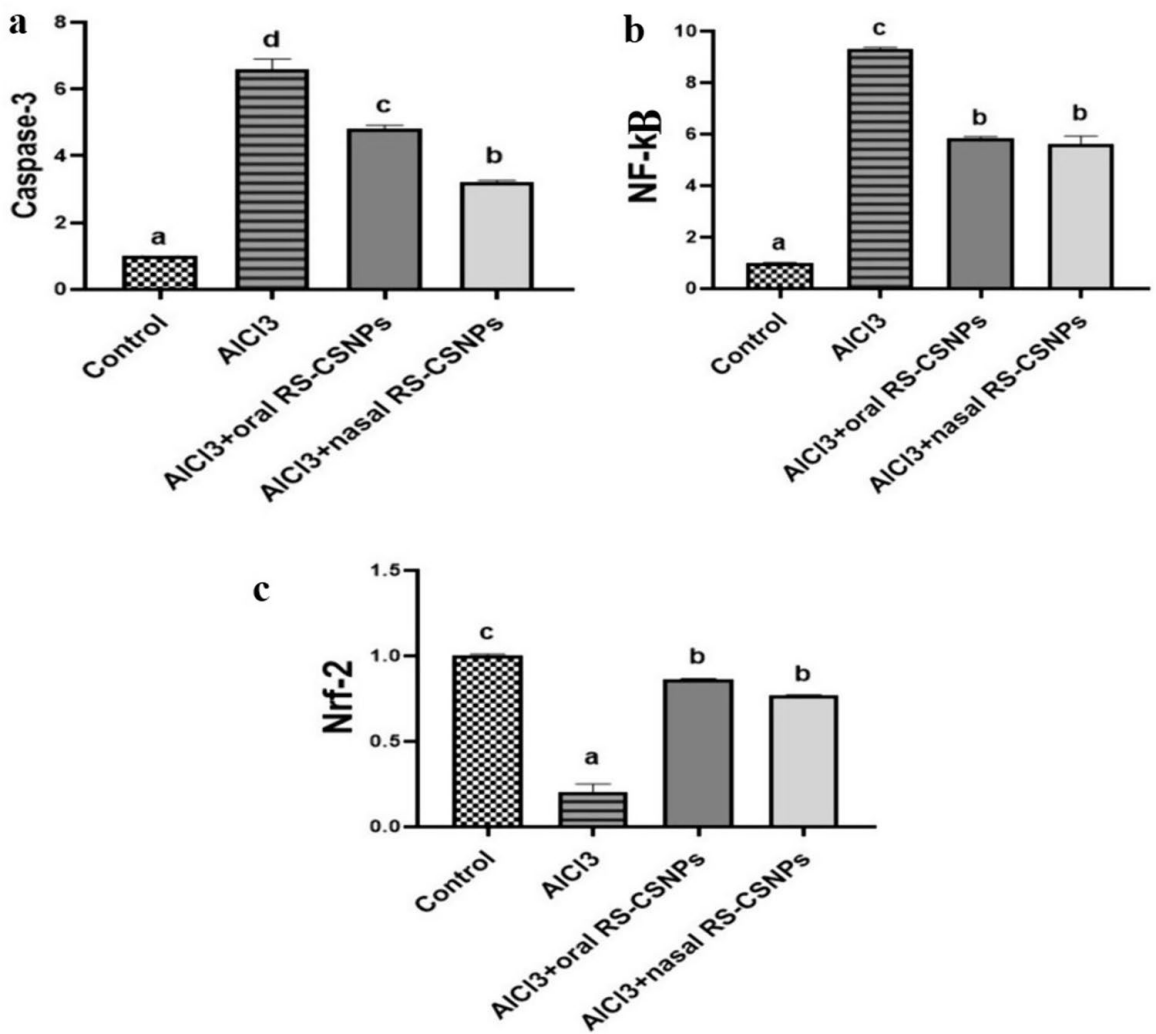
Bar charts representing the fold changes of **a** Caspase-3, **b** NF-κB, and **c** Nrf-2 genes in the brain. Values are presented as mean ± SE, (*n* = 5) and different lowercase letters indicated significant differences between groups at *P* < 0.05

The results of the real-time PCR showed significant up-regulation in the expression of Caspase-3 and NF-κB genes in the AlCl_3_ group compared to treated and control group. The transcript fold change of Caspase-3 was significantly reduced in AlCl_3_ + nasal RS-CSNPs compared to AlCl_3_ + oral RS-CSNPs group while in NF-κB, there is no significant difference between them. There was a significant down-regulation in transcription fold change of Nrf-2 gene in AlCl_3_ group compared to other groups. Both AlCl_3_ + nasal RS-CSNPs and AlCl_3_ + oral group revealed increased Nrf-2 transcript level with no significant difference between them (Fig. 5).

## Discussion

This study used nanostructure-mediated drug delivery as a promising approach for management of AD in addition to the use of I\N route as new trend for treatment of nervous disorders. The treatment agents (Desperation of RS loaded in CSNPs) employed in our study had an ameliorating effect on the level of histopathology and molecular investigation when administered orally and intranasally. Several previous studies reported the mitigative effect of RS on induced Alzheimer’s-like disease in laboratory animals (Tayebati et al. 2004; Abdel-Aal et al. 2011; Gupta et al. 2021) as it is already an approved drug from FDA. On the other hand, a few other trials used RS-loaded nano-particles in the treatment of AD (Haider et al. 2018; Kaboli et al. 2023). Most of RS-loaded nano-particles formulae used in previous research were given S\C (Ismail et al. 2013), I\V (Basharzad et al. 2022), transdermal (Chauhan and Sharma. 2019), or I/N (Fazil et al. 2012; Bhanderi et al. 2021).

In this study, we used nano-capsulation as method of preparation to obtain the best delivery of the therapeutic agent to brain (Singh and Lillard. 2009). Chitosan has been known for its mucoadhesive and highly biodegradable properties, making it a valuable therapeutic option for neurological disorders (Malhotra et al. 2009). We added a hydrophilic polymer or a surfactant (Tween 80) to increase the half-life of NP as it is capable of masking the NPs from the reticuloendothelial system (RES) cells (Alam et al. 2012). The addition of a surfactant on the surface of the NPs also represents a suitable strategy to increase their passage from the BBB. In fact, it is demonstrated that the absence of the surfactant on the surface of the nano-colloids significantly decreases the transport of the particles to the CNS with, consequently, less pharmacological effect (Allen and Smith. 2001). Additionally, another proposed mechanism is that Tween 80 plays a role in the blockage of the efflux system, reducing the pump-off effect of the P-gp (permeability glycoprotein) inhibitors, and consequently a higher pharmaceutical drug concentration in the brain is achieved (Raja et al. 2015).

The dispersion of RS in the polymer matrices led to a gradual dissolution and release of the drug, which will be complete over 24 h. Furthermore, this system may reduce drug leaching in the brain and decrease peripheral toxicity (Lockman et al. 2004; Meena et al. 2021). In our study, the intranasal route is better than oral route in management of AD, as I/N route helps in delivering drug directly into the brain by passing BBB thereby enhancing drug concentration in the CNS (Rassu et al. 2016). In addition, we found that RS loaded in CSNPs given through I\N route reserve the mucociliary nasal mucosa. Unlike the I\N route, we found lower effect of oral RS-loaded nano-particles than the I\N one although the differences in the therapeutic effect of both routes in some points were insignificant. We think that the use of nano-capsulation and chitosan as nano-carrier may offer some protection to the RS-loaded nano-particles from destruction in the stomach. However, the oral RS-loaded nano-particles induce some side effects on the gastrointestinal tract and liver indicating that it is unsafe as I/N route.

The RS chitosan-loaded nanoparticle used in this study achieved an improvement in the induced AD-like model through up-regulation of Nrf-2, which is a master regulator of the antioxidant response in cells and required for oxidative stress reduction (He et al. 2020). Additionally, Nrf-2 activation increases autophagy function (De Plano et al. 2023). Therefore, it decreases neuronal cell death and the accumulation of Aβ and hyperphosphorylated tau characterizing the pathogenesis of AD. Hence, increased Nrf-2 function may be a novel way to mitigate AD pathology and related cognitive problems (Uruno et al. 2020).

The different pathways that contribute to neurodegeneration in AD are highly interconnected via continuous NF-κB activity in both neurons and glial cells (Jha et al. 2019). NF-κB level was significantly decreased in both treated groups, and recent research reported that inhibition or limitation of NF-κB activation in the nervous system mitigate neuronal damage and aid in AD management (Kong et al. 2020).

In the current study, there is up-regulation of caspase-3 and over tau expression in brain tissue of AlCl_3_ group compared to other groups. Recent studies suggest that caspases may play a role in tau pathology (Chung et al. 2001; García-Sierra et al. 2008; Ramalho et al. 2008). The inhibition of caspase-3 and tau protein aggregates observed in the treated groups was confirmed by increased cell viability in the brain tissue. However, the down-regulation of caspase-3 in the I\N group was more than oral group indicating the superior effect of I\N route than oral one.

We recommend further investigations due to the lack of dose–response effect and small number of animals being tested in the current study.

## Conclusion

Based on our findings, both the oral and intranasal administrations of rivastigmine-loaded chitosan nano-particles desperation alleviated the adverse progressive effects of AD induced by the AlCl_3_ in rats. The I/N route was more effective and safer than oral route. The therapeutic agents used improved the histopathology, regulated the expression of NF-κB, caspase-3 and Nrf-2 in addition to reducing Tau aggregation and neuronal loss, thereby improved the function of the brain.

## Data Availability

The datasets used during the current study are available from the corresponding author on reasonable request.
